# Supplementary material for: Host Defense Peptides LL-37 and Lactoferrin Trigger ET Release from Blood-Derived Circulating Monocytes
Source: Biomedicines. 2022 Feb 17;10(2):469. doi: 10.3390/biomedicines10020469 (PMC8962388; doi:10.3390/biomedicines10020469)
Supplement: Supplementary file 1 [file biomedicines-10-00469-s001.zip › biomedicines-1560369-supplementary-Figure S1.pdf]

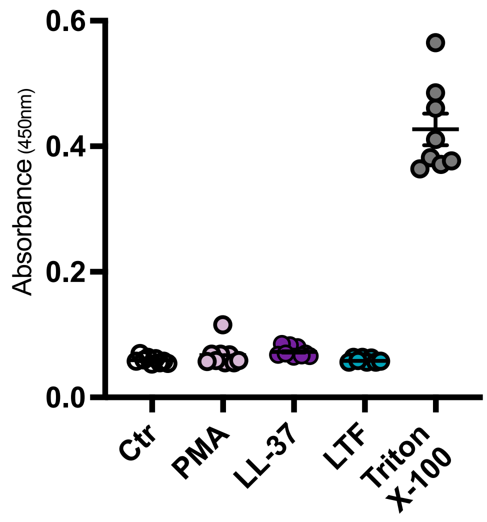

Supplemental Figure S1. Determination of possible cytotoxicity. Analysis of LDH release. Triton X-100 served as positive lysis control.
